# CIMS Detection of Hydroxymethyl Hydroperoxide: Insights into Alkene Ozonolysis in a Variety of Environments

**DOI:** 10.1021/acsestair.6c00053

**Published:** 2026-06-10

**Authors:** Andrew J. Lindsay, Khaled Shaifullah Joy, Lee V. Feinman, Kyle Banecker, Brigitte M. Weesner, Amalia Muñoz, Esther Borrás, Mila Ródenas, Teresa Vera, Rubén Soler, Ezra C. Wood

**Affiliations:** † Department of Chemistry, 6527 Drexel University, Philadelphia, Pennsylvania 19104, United States; ‡ 124153 Centro de Estudios Ambientales del Mediterráneo, Paterna, Valencia 46980, Spain

**Keywords:** hydroxymethyl hydroperoxide, ozonolysis, Criegee Intermediates, peroxides, formic acid, formaldehyde, chemical ionization mass spectrometry

## Abstract

Hydroxymethyl hydroperoxide (HMHP, CH_2_OHOOH) is produced from the reaction of water vapor with CH_2_OO, which is the simplest Criegee intermediate and is produced by the reaction of ozone (O_3_) with a terminal alkene. After formation, HMHP is thought to have a considerable lifetime upward of 1 day due to its reaction with OH, which produces formic acid and formaldehyde. HMHP measurements can therefore provide meaningful insight into atmospheric oxidation processes, especially alkene oxidation. We demonstrate the ability of iodide adduct chemical ionization mass spectrometry (I^–^CIMS) to detect HMHP and show data from three measurement studies that span a range of chemical environments. Measurements of HMHP are presented from a controlled chamber-based biomass burning (BB) O_3_ oxidation experiment, from outdoor sampling periods in Boise, Idaho, during the summer with mixed rural/biogenic influences, and from Philadelphia during summer and late fall during time periods of BB impact. Ambient HMHP concentrations ranged up to 100 pptv during summer at both field sites but rarely exceeded 10 pptv in the late fall. HMHP is often but not always correlated with OH oxidation products detected by I^–^CIMS, including I­(C_5_H_10_O_3_)^−^ from isoprene hydroxy hydroperoxides (ISOPOOH) and isoprene epoxydiols (IEPOX), providing insight into the relative importance of O_3_ versus OH oxidation and alkene composition. The greatest observed HMHP mixing ratios exceeded 300 pptv during an extended BB episode at the Philadelphia site caused by the historic Canadian wildfires of 2023 and were considerably greater than the chamber-simulated BB experiment. Given the age of the sampled air masses, these elevated concentrations suggest sustained production and long-range transport of HMHP, highlighting that HMHP can accumulate, persist, and act as an important precursor to its degradation products over long distances. Given how commonplace I-CIMS measurements have become in the past decade, measurements of HMHP may serve as a useful tool for assessing the budgets and chemistry of several important tropospheric compounds, including Criegee intermediates, formaldehyde, and formic acid.

## Graphical abstract

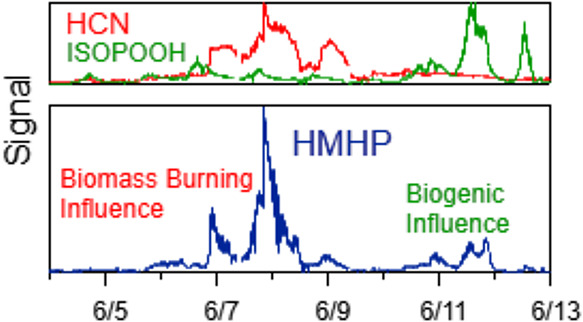

## 1 Introduction

Ozonolysis is a major sink for alkenes in the atmosphere and produces a range of oxygenated organic compounds, including carbonyls (e.g., formaldehyde, HCHO), carboxylic acids (e.g., formic acid, HCOOH), and hydroperoxides such as hydroxymethyl hydroperoxide (HMHP; CH_2_(OH)­(OOH)). Alkene ozonolysis is also an important nonphotolytic source of the hydroxyl radical (OH) which is the atmosphere’s primary oxidant that determines the atmospheric lifetime of most trace gases. HMHP is mainly formed from the ozonolysis of terminal alkenes, especially the abundant biogenic compounds isoprene, monoterpenes (e.g., β-pinene, limonene), terpenoids (e.g., linalool), and sesquiterpenes (e.g., β-caryophyllene). Because of its formation pathway, HMHP serves as an indicator of alkene oxidation and ozonolysis.^1^

An abbreviated reaction scheme for terminal alkene ozonolysis and subsequent HMHP formation is depicted in Figure 1. The initial alkene-ozone (O_3_) reaction forms a primary ozonide (not shown) that decomposes to produce a carbonyl and a carbonyl oxide (Criegee intermediate, CI) that has zwitterionic (or to a lesser extent, biradical) structure. A portion of these nascent CIs are vibrationally excited and either decompose, forming variable amounts of OH, HO_2_, and organic peroxy radicals, or undergo collisional stabilization that yields the stabilized CI (sCI).^2^ Nascent sCIs are also directly formed from the decomposition of the primary ozonide (i.e., without collisional cooling).^3–8^ The yield of sCIs from alkene ozonolysis varies considerably, with values as high as 60% for some alkenes.^3^ Formaldehyde oxide (CH_2_OO) is the simplest CI species and is formed by the ozonolysis of terminal alkenes (Figure 1). The dominant sink of stabilized CH_2_OO under most atmospheric conditions is its bimolecular reaction with the water dimer ((H_2_O)_2_),^2,9–11^ forming HMHP with a yield of 0.40.^2^ Reaction with the H_2_O monomer is also important, becoming competitive with the (H_2_O)_2_ reaction under very dry conditions (e.g., < 10% RH), and has a higher HMHP branching yield (0.73) than the (H_2_O)_2_ pathway.^2^ Alternative chemical sinks of the CH_2_OO sCI are mostly minor but include reactions with SO_2_,^12,13^ NO_*y*_,^13,14^ volatile organic compounds,^13,15–17^ and methane sulfonamide.^18^

**Figure 1:**
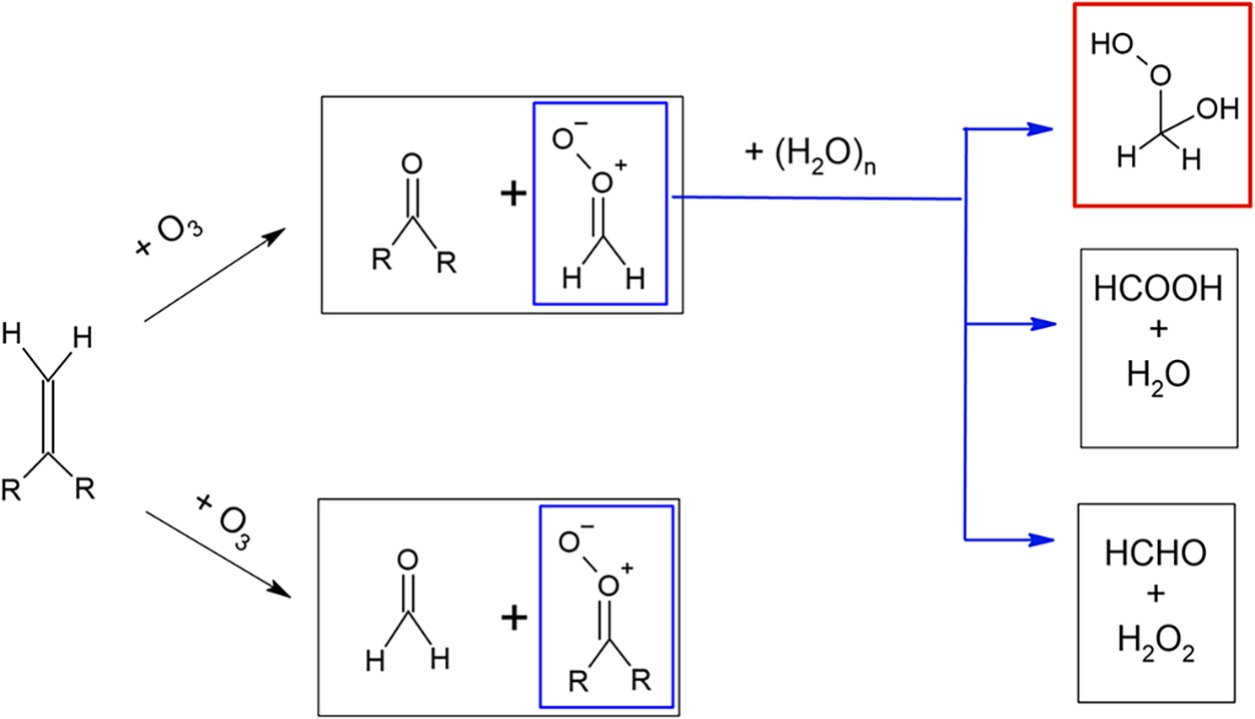
Hydroxy methyl hydroperoxide (HMHP) formation from ozonolysis of terminal alkenes. The initial reaction of ozone leads to a primary ozononide (not shown), cleaves the C–C double bond, and yields Criegee intermediates (blue boxes) and carbonyls. A portion of the Criegee intermediates undergoes collisional stabilization (not shown). The bimolecular reaction of the Criegee intermediate CH_2_OO with (H_2_O)*_n_* yields HMHP (red box) and other products. (H_2_O)*_n_* represents the water monomer (*n* = 1) and water dimer (*n* = 2);their reactions with CH_2_OO are competitive and yield different amounts of HMHP.

The rate constant for the reaction of HMHP with OH (*k* = 7.1 × 10^–12^ cm^3^ molecules^–1^ s^–1^)^1^ leads to a considerable lifetime of ∼20 h (for [OH] = 2 × 10^6^ molecules cm^–3^). The reaction proceeds by abstracting a hydrogen. Abstraction of the hydroperoxy and alcohol hydrogens primarily yields HCHO ([Disp-formula eq1]), whereas abstraction of a methyl hydrogen yields HCOOH ([Disp-formula eq2]).^1^

R1 $${\mathrm{CH}}_{2}\left(\mathrm{OH}\right)\mathrm{OOH}+\mathrm{OH}\to \mathrm{HCHO}+{\mathrm{HO}}_{2}+H_{2}O$$

R2 $${\mathrm{CH}}_{2}\left(\mathrm{OH}\right)\mathrm{OOH}+\mathrm{OH}\to \mathrm{HCOOH}+\mathrm{OH}+H_{2}O$$

Deposition of HMHP represents another important removal mechanism since its dry deposition rates are similar to that of nitric acid.^19^ Given its high Henry’s law coefficient (*K*_h_ = ∼ 1.7 × 10^6^ M atm^–1^ at 25 °C),^20,21^ wet deposition is likely a significant removal process as well. Heterogeneous reactions have also been suggested as important removal processes but are insufficiently characterized.^22,23^ HMHP photolysis is negligible under atmospheric conditions.^24,25^

There are alternative HMHP chemical sources (i.e., besides alkene ozonolysis). Oxidation of the methyl peroxyl radical CH_3_OO by both OH or atomic chlorine forms CH_2_OO, the precursor CI to HMHP, although with a low yield.^26^ Oxidation of formaldehyde by HO_2_ forms the hydroxy methyl peroxy radical CH_2_(OH)­OO·, and subsequent reaction with HO_2_ forms HMHP.^2,27^ Aqueous phase HMHP_(aq)_ also has an important role for atmospheric oxidation chemistry. HMHP_aq_ is produced via the addition of aqueous hydrogen peroxide (H_2_O_2,aq_) to HCHO_aq_.^28^ In clouds, this HMHP_aq_ rapidly oxidizes dissolved sulfur dioxide to sulfate while also regenerating HCHO_aq_. HMHP therefore plays an important role in sulfur particulate matter formation.^29^

Measuring HMHP is useful since it is a key intermediate that links the ozonolysis of terminal alkenes to simple oxygenated products (HCOOH, HCHO, H_2_O_2_), and can help to quantify the budget of sCIs. Despite its chemical relevance and considerable lifetime, there have been few atmospheric measurements of HMHP. Measurements of HMHP by CF_3_O^–^ CIMS in three different areas high in biogenic VOC emissions in the US in the Summer indicated concentrations typically in the hundreds of pptv and at most 1 ppbv.^1,2,19^ These measurements of HMHP by CF_3_O^–^ CIMS have been invaluable, though to our knowledge have been limited solely to the CalTech research group.

Current chemistry mechanisms employed by models typically assume rapid conversion of the CI precursor, CH_2_OO, to its eventual products like HCHO and HCOOH, bypassing the intermediate HMHP altogether. This includes, for example, the master chemical mechanism.^30,31^ Additionally, CH_2_OO stabilization yields are greatly simplified in models. The master chemical mechanism, for example, uses a near lower boundary CI stabilization branching yield of 0.37 for CH_2_OO while literature CI stabilization yields range beyond 0.60 and are dependent on the precursor alkene.^3^ The explicit inclusion of HMHP and the proper handling of CI stabilization will alter model outputs of relevant oxidation products, while a base mechanism (i.e., prompt CI conversion without HMHP) may act as an upper limit for HMHP degradation associated products. One model that was updated to explicitly include HMHP revealed profound reductions in HCOOH outputs of 10–90% compared to the standard model (i.e., prompt HMHP conversion).^9^ This is particularly important since several model studies have demonstrated standard models to underestimate HCOOH in both biogenic and biomass burning (BB) influenced environments.^22,32,33^

In this manuscript, we present real-time measurements of HMHP using iodide adduct chemical ionization mass spectrometer (I^–^CIMS), including chamber experiments and ambient observations to investigate HMHP formation, alkene oxidation, and O_3_ chemistry. Given how commonplace I^-^ CIMS measurements have become in the past decade, measurements of HMHP may be useful for assessing the budgets and chemistry of primary alkenes, Criegee intermediates, formaldehyde, and formic acid.

## 2 Methods

### 2.1 Site Information and Data Collection

HMHP and supporting species were measured at two field sites: Meridian, Idaho; and Philadelphia, Pennsylvania. Chamber experiments were conducted at the European Photoreactor (EUPHORE) facility in Valencia, Spain.^12^ HMHP was measured at all locations using I^–^CIMS (section 2.2).

Ambient sampling in Meridian, Idaho, was conducted in August 2019 approximately 400 m north of a major interstate highway and 10 km west of Boise. The location was influenced by agricultural lands located to the northwest, west, southwest, and south. The sampling configuration and further detailed information are described elsewhere.^34,35^ Supporting instruments used during this experiment include a cavity attenuated phase shift (CAPS) NO_2_ spectrometer^36^ and Teledyne API analyzers for NOx, CO, and O_3_ (models T200u, T300u, and T400) that were operated by the Idaho Department of Environmental Quality.

Measurements in Philadelphia were performed at a Drexel University campus building (39.954427° N, 75.187553° W) in June 2023 and November 2024. Instruments were housed in a laboratory approximately 30 m above ground level, and sampling inlets were extended outside of a window approximately 0.5 m from the building walls. The sampling location is in a dense urban setting, approximately 0.5 km west of Interstate 76, and approximately 0.25 km west of a commuter rail station and freight train tracks. Philadelphia also features urban forests and parklands with significant tree cover.^37^ Both sampling periods were affected by BB. First, aged smoke (>1 day) from the historic 2023 Canadian wildfires influenced the site during June 2023. Second, fresh smoke (aged <6 h) from New Jersey wildfires located ∼ 30 km to the east impacted the site during November 2024. Source attribution and plume age estimates were derived from fire detections retrieved from NOAA Hazard Mapping System^38^ and Fire Information for Resource Management System^39^ combined with NOAA air resources laboratory hybrid single-particle Lagrangian integrated trajectory (HYSPLIT) back trajectories,^40^ following previously used methods.^41,42^ Representative back trajectories during BB influenced periods are shown in the SI. Supporting instruments for Philadelphia measurements included a 2B model 205 O_3_ monitor, a Teledyne chemiluminescence NOx analyzer (Model T200u), and a LICOR CO_2_ and H_2_O analyzer (model LI-850).

For the ambient air sampling, most instruments sampled from a common inlet manifold.^34,43^ Briefly, the inlet was comprised of an initial sample tube that connected to a series of tees. The initial tee was connected to a diaphragm pump that ensured rapid transport of the initial sample tubing to the manifold. Subsequent tees were connected to instrument specific sample lines or connected to periodically automated lines designed for instrumental zeros or standard additions by delivering excess background air (cylinder zero air) or small flows of known concentration for some analytes (e.g., NO_2_ for the CAPS, acetic acid for the CIMS).

The EUPHORE facility and associated instrumentation are described in detail elsewhere.^44,45^ The EUPHORE reactor consists of a hemispherical fluorine–ethene–propene (FEP) polymer chamber with a volume of 200 m^3^. The chamber is equipped with large horizontal and vertical fans to ensure rapid and homogeneous mixing, typically achieved within approximately 2 min. In addition to the chemical ionization mass spectrometer (CIMS) described in the next section, a proton-transfer-reaction time-of-flight mass spectrometer (PTR-ToF-MS, IONICON) was deployed to measure the gas-phase chemical composition, while O_3_ concentrations were monitored using an O_3_ analyzer (Serinus 10, Ecotech).

Prior to EUPHORE experiments, the chamber was flushed and filled with clean air. The main experiment of interest from 7 November, 2024 had a relative humidity set to 40% and involved BB emissions sourced from pine wood that was combusted using a wood stove. The BB smoke was introduced into the chamber for 1 min during the flaming phase, which was identified by visible flames, to simulate fresh emissions. The experiment was performed under dark conditions, at ambient temperatures ranging between 294 and 296 K.

### 2.2 Chemical Ionization Mass Spectrometry

Two chemical ionization time-of-flight mass spectrometers (CI-ToF-MS; ARI/ToFWERK AG) were used in this work, both operating in similar fashion using iodide (I^–^) as a reagent ion. The ambient data and calibration experiments were made using the Drexel CIMS instrument which was equipped with a custom ion molecule reaction chamber (IMR) that is internally coated with PTFE.^34,46,47^ A second, near-identical CIMS instrument that was equipped with a Filter Inlet for Gases and Aerosols (FIGAERO) inlet^48^ and a commercial IMR^49^ was used for the EUPHORE chamber experiments.

Both IMR designs included a main sample inlet and a port through which the reagent ion was introduced. Reagent I^–^ was introduced in the reagent inlet by subjecting CH_3_I in N_2_ to a polonium ionizer. Analytes (e.g., HMHP and HCOOH) sampled through the main sample inlet interact with I^–^ (and I­(H_2_O)^−^) to form iodide-analyte adducts (e.g., I­(HMHP)^−^ and I­(HCOOH)^−^). The custom IMR (on the Drexel CIMS) has an additional port for humidified N_2_ to stabilize [H_2_O] during sampling. For the EUPHORE CIMS, the CH_3_I/N_2_ gas was humidified by bubbling through water prior to exposure to the Po ionizer. Generated ions are then transmitted from the IMR through two quadrupole ion guides and into a time-of-flight mass analyzer where they are separated by mass-to-charge ratio (*m*/*z*) and detected. The Drexel IMR lacked thermal control; corrections for this are described in the SI.

The CIMS IMR pressure was held at 70 mbar for the Meridian, Idaho, experiment, 80 mbar for both Philadelphia-based experiments, and 100 mbar for the EUPHORE CIMS. For the Drexel CIMS, inlet flow rates were ∼1.8 SLPM for the main sample inlet, ∼1.8 SLPM for the reagent ion inlet, and 0.5 SLPM for the humidified N_2_ inlet. These sample flow rates were maintained by stainless-steel critical orifices. Ambient air was sampled through 3 m of 0.64 cm ID PTFE tubing at ∼80–100 mbar, leading to a short residence time of 200 ms for the Drexel CIMS. The EUPHORE CIMS sampled chamber air via 1.5 m of 4 mm ID PFA tubing with a flow rate of 2 SLPM, leading to a residence time of 0.6 s. Further details of the sampling manifolds have been described previously.^34^

The mass resolving powers attained by the CIMS instruments were 4000 for the Idaho measurements, and ∼6000 for the Philadelphia and EUPHORE experiments, as determined using the Tofware analysis software (formerly ToFWERK AG, currently Aerodyne Research, Inc.). This software was used to isolate analyte ion peaks from other similar mass ions by assigning ion chemical formulas, using characterized parameters for peak shape and peak width, and accounting for mass calibration. The computationally separated peaks were integrated. Ions at the same unit mass as I­(HMHP)^−^ (191 *m*/*z*) are I­(H^15^NO_3_)^−^ (associated with the particularly abundant I­(HNO_3_)^−^ ion) and I­(SO_2_)^−^, which is occasionally present during BB-influenced periods. I­(HMHP)^−^ was specifically detected at 190.92106 *m*/*z* and would require a mass resolving power of ∼8200 for separation at fwhm and ∼18,800 for separation at full width at tenth of maximum (FWTM) from I­(H^15^NO_3_)^−^ (190.89770 *m*/*z*). The magnitude of the I­(H^15^NO_3_)^−^ peak is constrained by the neighboring I­(H^14^NO_3_)^−^ peak and the isotopic abundance of ^15^N. The instrumental peak shape is also well characterized based on numerous peaks in the mass spectrum that stem from a single contributing ion. As a result, these multiple peaks were effectively deconvoluted by the Tofware software. A representative mass spectrum depicting this deconvolution is included in the SI (see Figure S4).

#### 2.2.1 Calibration

CIMS analyte-ion signals were converted to concentration using experimentally characterized sensitivity values. Calibration experiments were performed by delivering known analyte concentrations to the IMR and monitoring the signal response. These ion signals (in counts/s) were normalized to 10^6^ counts per second (cps) signal of total reagent ions (considered as the sum of I^–^ and I­(H_2_O)^−^) to account for variability or drift in reagent ion abundance. Sensitivities are therefore expressed in units of ncps pptv^–1^.

The sensitivity of CIMS to analytes is also generally dependent on the absolute water vapor concentration within the IMR ([H_2_O]_IMR_).^50^ We therefore spanned experimental calibrations over a range in [H_2_O]_IMR_ to characterize sensitivity-[H_2_O]_IMR_ relationships. These relationships are used in conjunction with real time [H_2_O]_IMR_ values, typically measured using a Vaisala HMP60 probe positioned at the IMR exhaust, to account for humidity caused variability in CIMS sensitivity. Dependence on [H_2_O]_IMR_ is further detailed in the SI (Section S2.2). Unstable IMR temperature can also influence CIMS sensitivity, which unstable temperatures during the Meridian measurements were accounted for using sensitivity dependences on temperature of −3 to −8% °C for various analytes (detailed in SI Section S2.2).

#### 2.2.2 Standard Calibrations

Besides HMHP, relevant compounds for the ambient sampling that were calibrated (using the Drexel CIMS) include hydrogen cyanide (HCN), formic acid, and acetic acid. Typical sensitivity values (at [H_2_O]_IMR_ = 7 ppthv) were 0.035, 3.0, and 0.13 ncps pptv^–1^, respectively. HCN was calibrated using a standard gas cylinder (5 ppm in N_2_; Cal Gas Direct),^43^ while the organic acids were calibrated using fresh laboratory-built permeation tubes. Automated periodic permeation tube additions to the inlet manifold provided real-time sensitivity tracking for the Meridian, ID, experiment, while laboratory-characterized sensitivity-[H_2_O]_IMR_ relationships (obtained using the Drexel CIMS) were used for other data sets.^34^ For uncalibrated species of interest (e.g., ISOPOOH), the dependence of the sensitivities on humidity were estimated using the acetic acid sensitivity-[H_2_O]_IMR_ relationship, which is generally representative of numerous compounds,^34,50^ and scaled to a median sensitivity of 1 ncps pptv^–1^. For the EUPHORE CIMS, we applied an effective sensitivity to HCN of 0.047 ncps pptv^–1^ based on the normalized I­(H_2_O)^−^ signals since [H_2_O]_IMR_ was not directly measured.

#### 2.2.3 HMHP Calibration

Gaseous HMHP was generated by mixing ethene (C_2_H_4_) with O_3_ under controlled conditions and a known reaction time. The C_2_H_4_ + O_3_ reaction yields CH_2_OO*, which subsequently undergoes collisional cooling and reacts with (H_2_O)_n_ to generate HMHP (see Figure 1). This calibration method is relatively easy, safe, and straightforward to perform, but suffers from the high uncertainties regarding the sCI yields. For reference, alternative HMHP procedures involve bubbling formaldehyde vapor through a hydrogen peroxide or derivative (e.g., urea hydrogen peroxide) solution.^19,24^ Photolytic methods are possible, including the photolysis of formaldehyde^2^ and the photolysis of CH_2_I_2_ to produce the related CI CH_2_OO.^11^

A schematic of the calibration setup and representative flow rates is shown in Figure 2. Known concentrations of O_3_ (2B model 205 O_3_ calibration source) and C_2_H_4_ (610.4 ± 12.2 ppmv; diluted to desired concentration) were introduced to a new 3.05 m long, 0.95 cm ID PTFE reaction chamber. Excess flow from the O_3_ source was vented prior to the C_2_H_4_ mixing tee and reaction chamber. This exhaust was monitored by a LICOR CO_2_ and H_2_O instrument. Following the reaction chamber, a dilution tee introduced a set N_2_ flow with variable humidity. The final mixture was delivered to the IMR.

**Figure 2:**
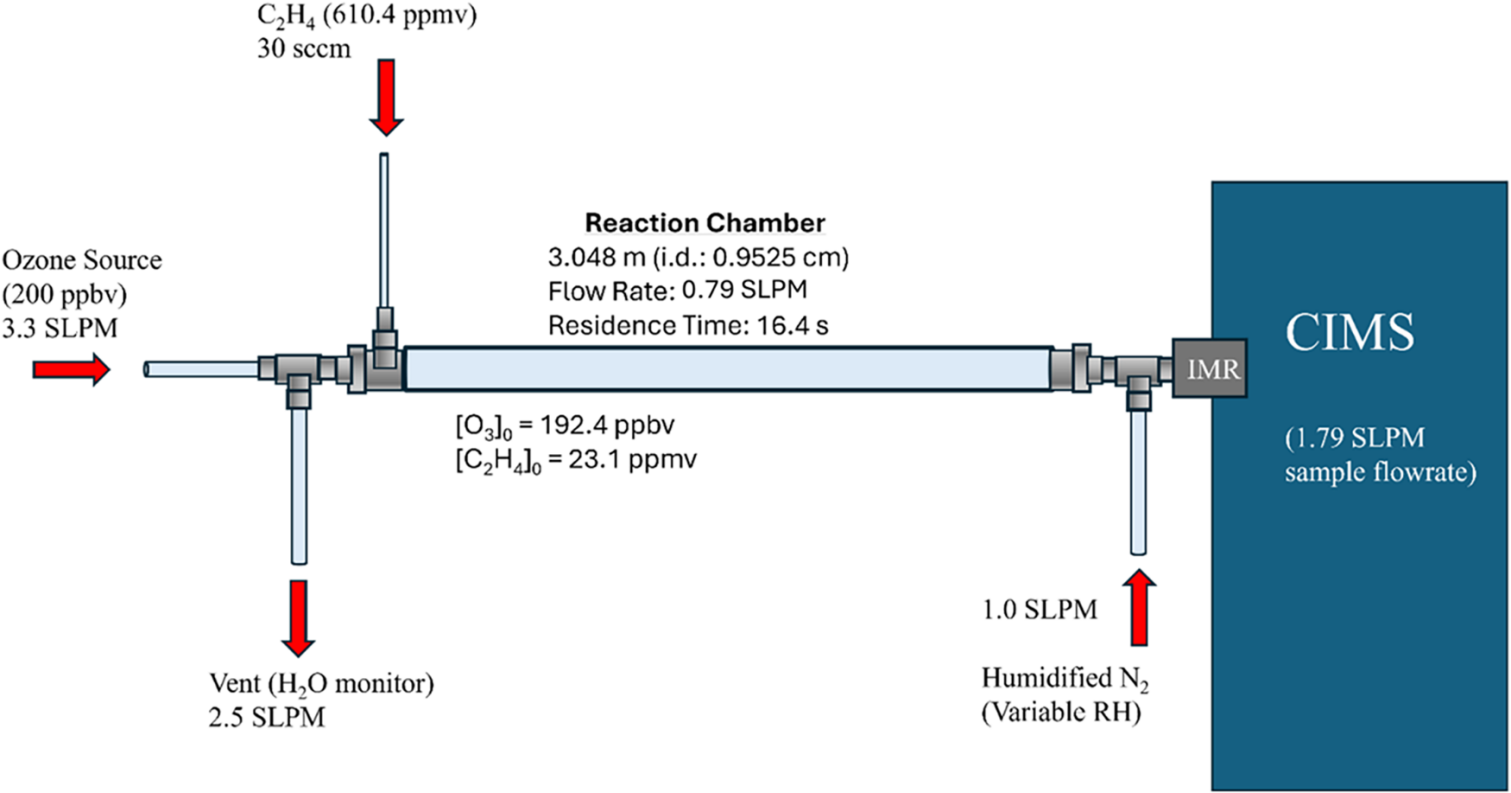
Plumbing schematic for HMHP calibration experiments. Known amounts of O_3_ and C_2_H_4_ are delivered to a reaction chamber where HMHP is generated for a known amount of time, then diluted to vary sample humidity, and transmitted to the CIMS IMR inlet. Schematic not drawn to scale.

The total flow rate in the reaction chamber for a representative calibration experiment was 0.79 SLPM (18.8 cm s^–1^) corresponding to a 16.4 s residence time. The initial reaction conditions ([O_3_] = 192.4 ppbv; [C_2_H_4_] = 23.1 ppmv; [H_2_O] = 5.0 ppthv; *T* = 296.4 K; *P* = 1007.4 mbar) produced 560 pptv HMHP after 16.4 s, which was subsequently diluted to 247 pptv and delivered to the IMR. The HMHP yield was quantified using the kinetics of the rate-limiting step (C_2_H_4_ + O_3_; *k*_C2H4+O3_ = 1.7 × 10^18^ at 298 K) and an effective HMHP yield of 19%. This 19% yield combines the sCI yield (0.42) for C_2_H_4_ ozonolysis^3^ with a weighted 0.45 HMHP formation yield form CH_2_OO + (H_2_O)*_n_* reactions. The water dimer (H_2_O)_2_ concentration was calculated using a dimer–monomer equilibrium constant (i.e., 2H_2_O (g) ⇌ (H_2_O)_2_ (g)) of 0.05 atm^–1^.^11,51^ For reference, use of the rate-determining step in conjunction with the overall HMHP yield is deemed appropriate since HMHP outputs are in agreement with a kinetic simulation that accounts explicitly for additional steps of CH_2_OO* stabilization and CH_2_OO + (H_2_O)*_n_* reactions (Section S2.3 of SI).

The representative HMHP calibration was performed over a 3.0–7.0 ppthv range in [H_2_O]_IMR_, and results are shown in Figure 3. Sensitivity values decreased from ∼14 to 7 ncps pptv^–1^ with increasing humidity. Typical [H_2_O]_IMR_ values during ambient data sampling are between 6–8 ppthv, corresponding to sensitivities of 4.6 and 7.1 ncps pptv^–1^. In contrast, voltage scanning experiments^52^ predicted a sensitivity of only ∼0.5 ncps pptv^–1^. Use of this much lower sensitivity would have produced unrealistically high HMHP concentrations during the ambient sampling and underscores the importance of conducting true calibrations when the sensitivity of individual compounds is needed.

**Figure 3:**
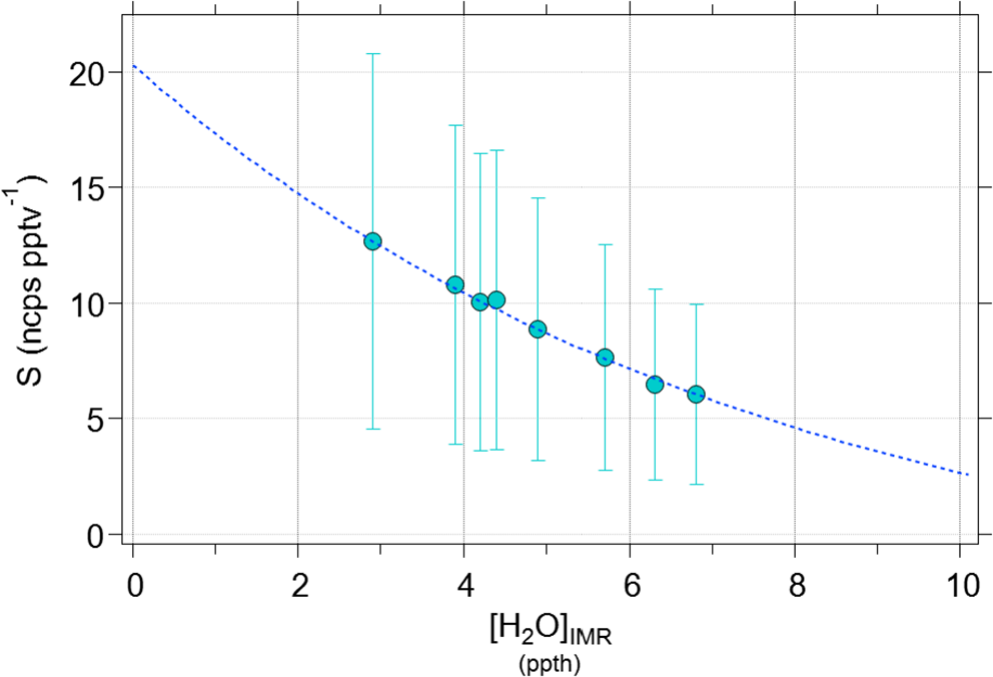
HMHP sensitiviy as a function of IMR humidity obtained from a representative calibration experiment. Error bar boundaries of individual calibration data points were set using the 64% accuracy. The calibration data was fit to an exponential function as described in the Supporting Information (Section S2.2).

We ascribe a 64% uncertainty (2σ) to the quantified concentration of calibrant HMHP delivered to the IMR. This value was generously estimated and accounts for error in the following factors: the C_2_H_4_ + O_3_ rate coefficient (10%), the initial O_3_ and C_2_H_4_ concentrations (5% each), the 0.19 mechanism yield (62%), and an additional 2% error corresponding to a possible 0.3 s inaccuracy in the 16.4 s reaction time. The combined error is dominated by the 62% mechanism yield uncertainty. Further details regarding these uncertainties are included in the SI (Section S2.3).

We include an additional 25% uncertainty for reported HMHP concentrations during ambient sampling to account for potential drift in the sensitivity to HMHP between the laboratory calibration and field sampling, and possible irreversible sampling losses which were likely less than 10% due to the low residence times in the sampling tubing. Reversible losses of HMHP were negligible, evident by the fast time response in the HMHP signal when concentrations were abruptly increased or decreased during calibrations or when switching between gas and particulate mode using the FIGAERO inlet (i.e., in contrast to what is observed for “sticky” compounds like nitric acid). The final accuracy in HMHP concentrations is 69% for the ambient data collected by the main CIMS instrument. Since the EUPHORE chamber experiments involved a separate CIMS instrument that had operational differences, including different voltage settings, a different IMR, and a different way of determining the IMR humidity, we assign a greater uncertainty to those measurements (factor of 2). Since we are not comparing our measured HMHP mixing ratios to those predicted by a 3-D model (for example), these relatively high uncertainties do not affect the main findings presented in this manuscript. For future comparisons of measured to modeled HMHP mixing ratios, these uncertainty values would need to be significantly reduced.

The experiment presented in Section 3.1 had consistent I­(H_2_O)^−^ signals (relative to total reagent counts) during gas phase sampling, associated with [H_2_O]_IMR_ value of 5.5 ppth (further discussed in SI). We applied an effective sensitivity to HMHP of 8.6 ncps pptv^–1^. Typical signal-to-noise ratios for both instruments were at least 20 for the vast majority of the measurements.

## 3 Results and Discussion

### 3.1 Ozonolysis Chamber Experiment

Results for a chamber experiment focused on monitoring BB smoke subjected to O_3_ (∼40 ppbv) under dark conditions are shown in Figure 4. The chamber was initially void of smoke. After 60 min, smoke generated from flaming combustion of pine was introduced. This is evident by the increase in HCN, a tracer for BB smoke,^41,53^ up to near 1 ppbv and similar increases in the PTR-MS signals for benzene, toluene, and furan. Ozone was injected after 180 min of sampling and supplemented with a second injection at 285 min to return to a near 40 ppbv concentration.

**Figure 4:**
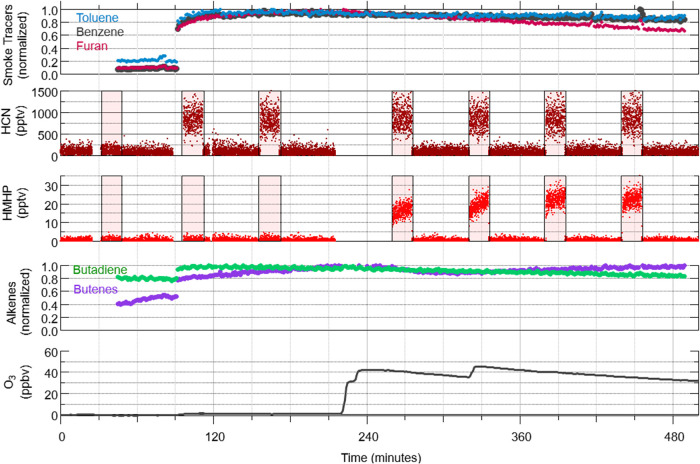
Chamber experiment data showing HMHP formation after exposure of BB smoke to O_3_ under dark conditions. The I^–^ CIMS measured HCN and HMHP (shown at a sampling frequency of 1 Hz) and was operated with a FIGAERO (Filter Inlet for Gases and Aerosols) inlet that modulates between sampling the gas (shaded regions) and particle-phases. Supporting observations are presented at 1 min frequency.

Concentrations of HMHP and HCN were measured using an I^–^CIMS with a FIGAERO inlet that modulates between sampling gas and particle phase. As expected, HMHP was observed only during gas phase sampling periods of the FIGAERO cycle.

HMHP values increased, exceeding 20 pptv, following the addition of O_3_ into the chamber, indicating interaction between O_3_ and BB-derived alkenes. Example alkene signals for butadiene and butene (i.e.,1-butene and 2-butene sum) are included in Figure 4 and show enhancements concurrent with smoke addition and steady signal after O_3_ introduction consistent with lengthy ozonolysis chemical lifetimes (several hours for butadiene, ∼1 day for 2-butene, and several days for 1-butene at 40 ppbv O_3_). All species are affected by a slow 3.1% h^–1^ chamber dilution rate, which explains the observed signal decay for chemically stable species like benzene.

Overall, this experiment acts as simple case for investigating HMHP formation from O_3_ chemistry and is analogous to fresh BB smoke plumes and BB influenced air masses under dark and low OH conditions (e.g., nighttime). The HMHP observed appears to reach a steady concentration, which is explained by a combination of continued formation and losses, including slow dilution, possible aerosol uptake, or reaction with any generated OH or NO_3_. HMHP production was observed for other experiments as well, including photochemical experiments in which no O_3_ was directly added. In all experiments, HMHP was only observed after O_3_ was either injected into or formed photochemically in the chamber.

### 3.2 Ambient Data

#### 3.2.1 Meridian, Idaho: Mixed Urban-Rural Site

HMHP measurements and supporting O_3_, NOx, CIMS results, and wind data during summer 2019 (8/14 to 8/22) at the Meridian, Idaho, mixed urban-rural site, are presented in Figure 5.

**Figure 5:**
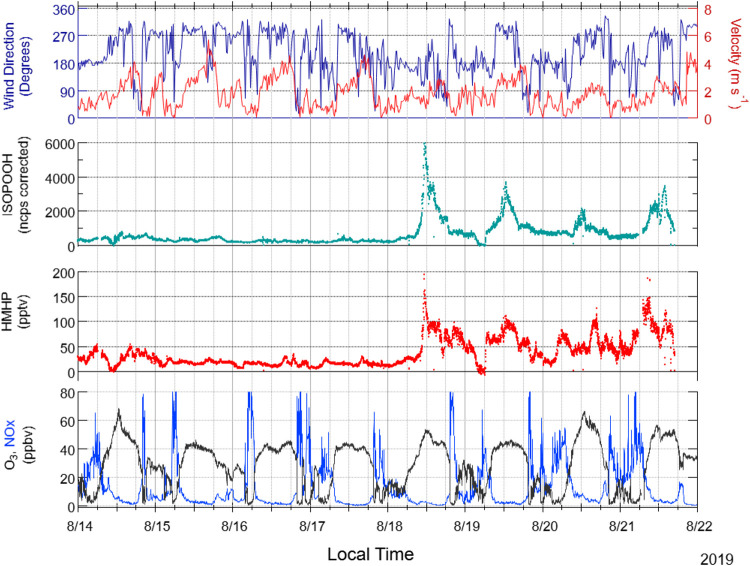
Ambient data collected during the summer 2019 sampling period in Meridian, Idaho. Data is presented on a 1 min averaged time basis.

For the period shown, HMHP concentrations remained below 50 pptv (average 15 pptv) for 4 consecutive days (8/14–8/17), exhibited a significant transition period (8/18), and remained elevated (average 50 pptv) with concentrations that occasionally exceeded 100 pptv for several periods. This shift from a clean, stable period corresponds with the observed transition in the wind direction - westerly flow dominated the first, low HMHP period, whereas slower southerly winds dominated the latter, high HMHP period of data. The change in both HMHP and wind indicates the transport of alkene-rich air masses to the ground site, likely from biogenic influence from surrounding rural areas and forests, though influence from a major highway located 400 m directly south is possible.

The general HMHP trend (i.e., a low concentration period, followed by a transition period, then a high concentration period) is generally consistent with the CIMS ISOPOOH signal trend. This further indicates the shift from minimal alkene influence to sustained alkene influence. ISOPOOH measured by the CIMS specifically represents the ion I­(C_5_H_10_O_3_)^−^ from both isoprene hydroxy hydroperoxides and isoprene epoxydiols that are produced by the OH-based oxidation of isoprene, therefore indicating biogenic influence. The ISOPOOH signals were corrected to account for changes in [H_2_O]_IMR_. Assuming an effective sensitivity of 1 ncps pptv^–1^, ISOPOOH concentrations were usually below 0.5 ppbv but at times exceeded 2 ppbv. These concentrations fall within the range of other ISOPOOH measurements in biogenic environments.^54,55^ The HMHP and ISOPOOH signals do not perfectly track each other. This may be explained by temporal variation in the relative rates of O_3_ based oxidation relative to OH-based oxidation, the relative lifetime of HMHP and ISOPOOH, and the presence of other terminal alkenes that generate HMHP (e.g., other biogenic alkenes and vehicle-emitted alkenes).

Ozone displayed a consistent pattern, with daytime peak values of 40–70 ppbv and often near-zero values during nighttime largely due to titration by vehicular NO emissions. The largest HMHP values observed did not align with the greatest O_3_ periods, consistent with HMHP concentrations being more sensitive to changes in air mass composition, specifically with respect to alkenes, rather than local photochemistry. Considering that its loss due to reaction with OH leads to a considerable lifetime (>20 h midday), the HMHP observed at the site was likely formed in transit during the preceding day or two. This is consistent with sustained HMHP concentrations of at least 25 pptv at night even though O_3_ concentrations were near zero. Of note is the low O_3_ period during the morning of 19 August, when [O_3_] was titrated to below 7 pbbv and both HMHP and ISOPOOH concentrations were extremely low (<5 pptv), suggesting there had been little O_3_ or OH oxidation of isoprene in the recent history of that air mass.

#### 3.2.2 Philadelphia, PA: Urban Site with BB Influence

Data collected in Philadelphia during the late fall and summer are shown in Figures 6 and 7, respectively. Concentrations of HMHP during the late fall of November 2024 were typically below 5 pptv. Concentrations during the summer period were considerably higher, as high as 700 pptv, though there were stable periods with HMHP below 100 pptv. The HMHP levels vary during both sampling periods, typically coinciding with other measured species associated with BB (i.e., HCN) or oxidation products (ISOPOOH and isoprene hydroperoxyl nitrate (IPN)).

**Figure 6:**
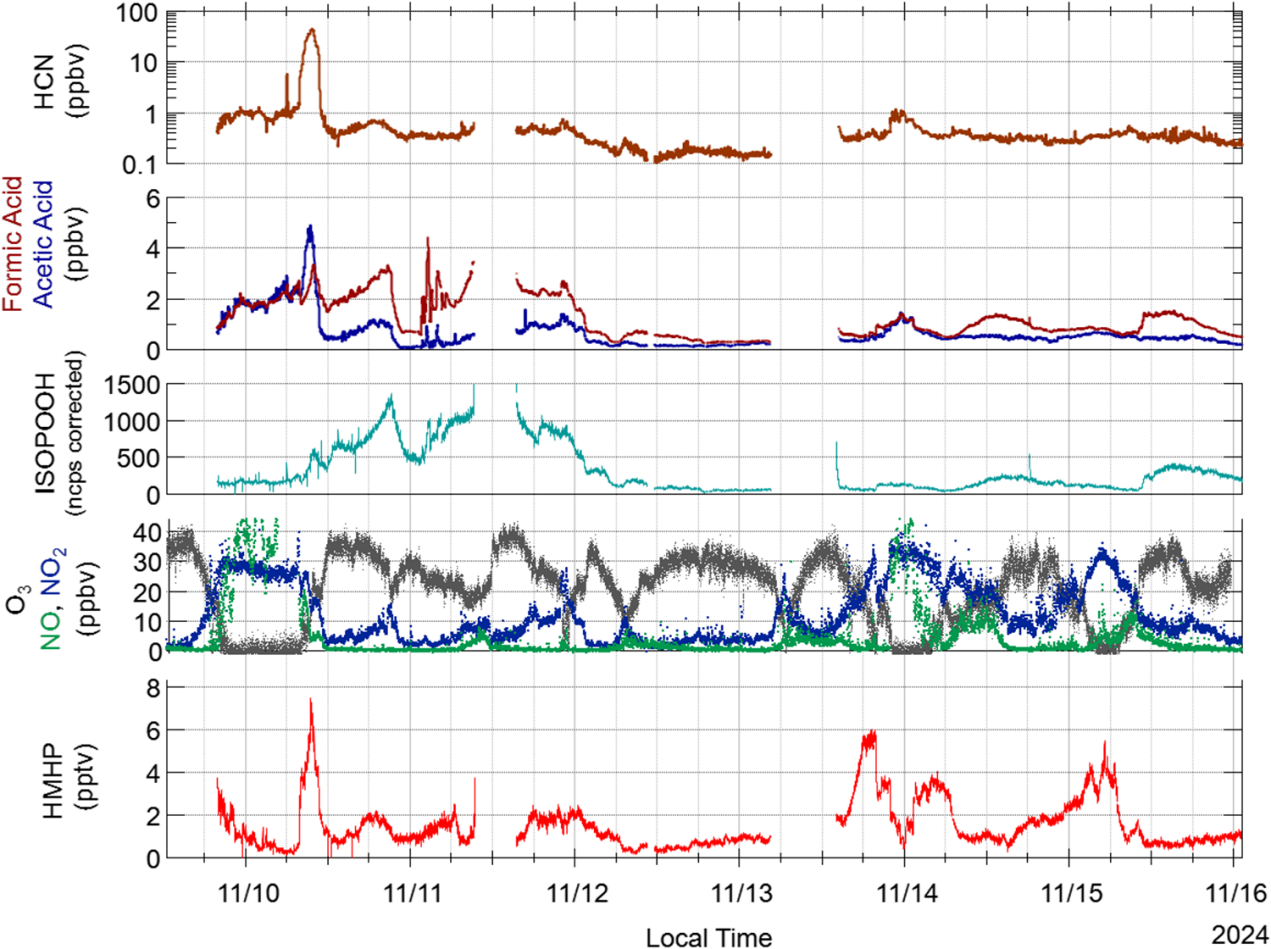
Ambient data collected during the late fall sampling period in Philadelphia. Note that HCN is plotted on a logarithmic scale. Data is presented on a 1 min averaged time basis.

**Figure 7:**
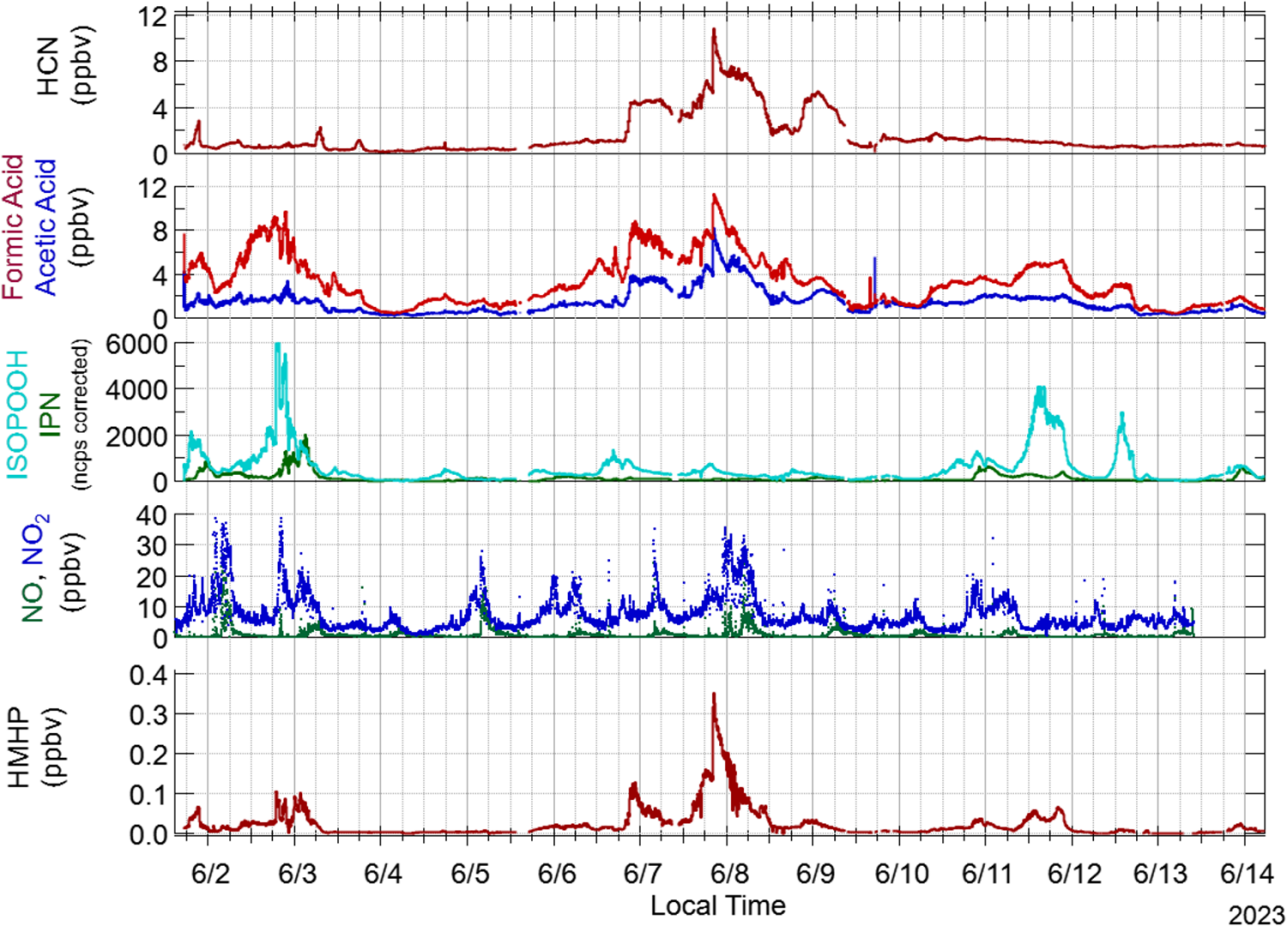
Ambient data collected during the summer period in Philadelphia. Data is presented on a 1 min averaged time basis.

The BB influenced periods, like the Section 3.1 chamber results, were indicated by elevated [HCN]. The highest HMHP concentrations across all data sets were observed in the summer during a multiday BB smoke episode between 6 June and 9 June 2023 from the historic 2023 Canadian wildfires (Section 2.1), evident by [HCN] exceeding 4 ppbv. Observed HMHP generally tracked the HCN temporal pattern during the BB episode, indicating that HMHP was associated with and was formed in the BB plume. The BB smoke was consistently aged >1.5 days, and considering the lifetime of HMHP for characterized loss mechanisms (OH-based removal lifetime is >20 h midday), HMHP was able to be generated within the plume in transit to the sampling site.

The fall measurement period was also impacted by BB smoke. This plume (11 November between 9:00 and 12:00 local time) had an approximate age of 6 h. Especially high HCN levels were observed, peaking near 40 ppbv and nearly 4 times higher than the greatest HCN values observed during the historic summer Canadian smoke event. These high HCN concentrations appear reasonable based on the measured 30 ppmv increase in [CO_2_], a modified combustion efficiency (MCE; Δ­[CO_2_]/(Δ­[CO_2_] + Δ­[CO])) of between 0.8 ^56,57^ (for smoldering combustion) or 0.9 (for flaming combustion), and a Δ­[HCN]/Δ­[CO] emission ratio between 3.9 ppbv ppmv^–1^ ^58^ and 12.4 ppbv ppmv^–1^.^56^

This BB temporal correlation is similarly observed in the fall period. HMHP concentrations were much lower than the summer values despite there being the greatest observed HCN values reaching up to 40 ppbv. This is likely due to the low background O_3_ concentrations and, most importantly, a muted biogenic influence (i.e., lower biogenic alkene load) required for HMHP production via alkene ozonolysis. Ozone remained nearly depleted at the Philadelphia site prior to the smoke presence, though this background O_3_ is likely not representative of the exposure of the smoke plume to O_3_ due to interactions with locally emitted NO (i.e., O_3_ was titrated) as evidenced by substantial NO_2_ concentrations. The smoke transit time to the sampling location was also considerably shorter than the 2023 summer, providing less reaction time.

During the late fall measurements, overall ISOPOOH levels were much lower than in the summer, consistent with lower OH concentrations and much lower isoprene emissions, which depend on temperature and sunlight. Enhancements in the ISOPOOH signal in the BB plume were minimal, suggesting a combination of limited emissions of isoprene from the fire and again, little OH oxidation. Episodes with enhanced HMHP during the non-BB periods (11/13 and 11/15) also lacked ISOPOOH indicating either low OH values or the importance of nonbiogenic alkenes (e.g., local urban emissions). Portions of these events have near-zero [O_3_] due to local Philadelphia NO emissions, which indicates the transport of regionally produced HMHP.

During the summer data, all episodes of elevated HMHP (both BB and non-BB) were concurrent with ISOPOOH observations. Notably, the relative amounts of HMHP to ISOPOOH appear somewhat consistent during non-BB periods. The BB episode, however, had much higher HMHP to ISOPOOH ratios, likely due to continuous production during plume transport, its long lifetime, persistent O_3_ concentrations, and production from terminal alkenes other than isoprene.

### 3.3 Discussion

HMHP concentrations lacked a diurnal pattern in all three of the ambient data sets. This is likely due to a combination of its long lifetime and the diurnal patterns of its precursors—O_3_ and terminal alkenes. Ozone itself has a lifetime of days, and concentrations rarely vary by more than a factor of 2 between day and night (aside from NO titration events that often occur in a shallow vertical layer near the surface at night). Biogenic alkene emissions, especially isoprene, are highest during the day, but many biogenic alkenes persist through the night as well. The coarse correlation between HMHP and isoprene oxidation products confirms the prevailing role of biogenic VOCs on its formation chemistry, leading to the lowest concentrations observed when ISOPOOH was also very low.

Observed ambient HMHP concentrations were generally in line with previously reported values.^19,2,1^ In the two summer data sets (Meridian and Philadelphia), HMHP concentrations were either comparatively low due to a lack of alkene-chemistry influence or high for periods sometimes exceeding 100 pptv. Both the low and high HMHP episodes often lasted for multiday periods. The late fall Philadelphia data set had lower observed HMHP values in comparison—peaking near 10 pptv—most likely due to a muted biogenic influence (i.e., lower temperatures, dormant forests).

HMHP enhancements coincided with the various BB events observed during the summer Philadelphia measurement period. Notably, the greatest HMHP values of all ([HMHP] = ∼400 pptv) were observed during significant BB influence from the historic 2023 Canadian wildfires. In contrast, only modest enhancements in HMHP were observed during the Philadelphia late fall BB event. The relevant O_3_–BB chamber experiment also had a lower observed HMHP enhancement of ∼25 pptv after several hours. This can be attributed to numerous factors, including higher ambient [O_3_] (chamber [O_3_] = ∼40 ppbv), sustained HMHP production over a longer period, mixing of other regional alkene emissions (e.g., mixing of fresh biogenic emissions, which were not present in the chamber study), and a different alkene emission profile.

Our findings support a considerable lifetime of HMHP. Concentrations remain elevated even with nearly depleted O_3_ values (e.g., nighttime Meridian, Idaho, results; the fall Philadelphia BB plume), indicating episodes of transported oxidation products rather than purely local photochemistry. As previously mentioned, HMHP is often assumed short-lived and is absent in common chemical mechanisms. Implementation of HMHP in chemical models will impact its degradation products of HCHO and HCOOH. Notably, several recent BB studies have reported model measurement discrepancies for HCOOH.^22,32,33^ For these instances, the explicit inclusion of HMHP into a chemical mechanism may act as a missing source of HCOOH since we have observed sustained concentrations in BB plumes. If changed to reflect a longer lifetime, then HCOOH model-measurement discrepancies may become more profound. Proper HMHP implementation also depends on knowledge of ozonolysis sCI yields, which currently appear challenging to accurately constrain since yields differ among alkenes. Further sCI research is necessary along with recommendations for HMHP implementation into chemical mechanisms. Finally, given that the I^–^CIMS technique is widely employed by the atmospheric chemistry community, the opportunity exists to greatly increase the number of HMHP data sets via analysis of existing and future measurements, which may in turn lead to an improved understanding of the global budgets of sCIs, HCHO and HCOOH.

## Supplementary Material



## Data Availability

EUPHORE environmental chamber data for the presented experiment can be found at https://data.eurochamp.org/data-access/chamber-experiments/#/datasets/2a855aa7-66fa-4cbd-aa2f-e161e82e5c2c.

## Notes

Details of the biomass burning events, CIMS peak fitting, CIMS calibration, and measurement uncertainties (PDF)

## §

Department of Chemistry, University of Colorado and CIRES, Boulder CO 80309, United States
