# Supplementary material for: CIMS Detection of Hydroxymethyl Hydroperoxide: Insights into Alkene Ozonolysis in a Variety of Environments
Source: ACS EST Air. 2026 Jun 10;3(7):1832–42. doi: 10.1021/acsestair.6c00053 (PMC13367238; doi:10.1021/acsestair.6c00053)
Supplement: Supplementary file 1 [file ea6c00053_si_001.pdf]

CIMS Detection of Hydroxymethyl Hydroperoxide: Insights into Alkene Ozonolysis in a Variety of Environments

Andrew J. Lindsay, Khaled Shaifullah Joy, Lee V. Feinman; Kyle Banecker; Brigitte M. Weesner; Amalia Muñoz; Esther Borrás; Mila Ródenas; Teresa Vera; Rubén Soler; Ezra C. Wood*

S1.0 Biomass Burning Events

Summer ambient data collected in July 2023 was impacted by the historic Canadian wildfires. Geographic wildfire and smoke data acquired from the MODIS Hazard mapping system[1] is presented in Fig. S1. Shown are active fires and biomass burning smoke present on 6 July, which coincides with period of ambient sampling with consistent HCN concentrations above 5 ppbv. For this selection of data, there are vast burn areas in Canadian and detected wildfire smoke that connects these fires to Philadelphia. Additionally, back trajectories acquired using the NOAA air resources laboratory hybrid single-particle Lagrangian integrated trajectory (HYSPLIT)[2] model were used to suggest the age of sampled smoke. HYSPLIT was computed using archived Global Data Assimilation System (GDAS) meteorological data (1 degree resolution) starting at the Philadelphia site from a 30 m sampling height and run 72 h backward in time. The resulting back trajectories collected over the course of the several day BB event consistently show Philadelphia air to be sourced from the north or north-northwest from areas with Canadian wildfires (Fig. S2). The trajectories consistently suggest smoke age of at least 1.5 days.

Fall ambient data in November 2024 features one biomass burning influenced period on 10 November in the early morning. A local news reports suggested New Jersey fires (located within 30 km) as responsible.[3] The local active wildfires are mapped in Fig. S3. Fire data was retrieved using the FIRMS (Fire Information for Resource Management System) interactive map focused

on the United States and Canada,[4] specifically from NOAA 20 and NOAA 21 satellite VIIRS (visible infrared imaging suite) measurements. HYSPLIT back trajectories for the morning with the BB episode are also mapped. The sampled air shifts from being sourced from the north (not shown), temporarily to the east and southeast, then towards the south. Trajectories during this shift from 4:00 and 8:00 hours (local time) are within the proximity of the two local wildfires at 6 or fewer hours backward in time, suggesting fresh, fewer than 6-hour aged smoke impacting the site. The trajectories heights from the sampling location (at height 100 m) are maintained near constant, indicating minimal vertical mixing which helps explain the especially high smoke tracer (HCN) observations that suggest a more dense plume sampled than the historic summer 2023 smoke event.

No other BB events were observed during the November 2024 sampling period. The fires were only active for several day periods, and the sample air was rarely sourced from this New Jersey region based on HYSPLIT runs.

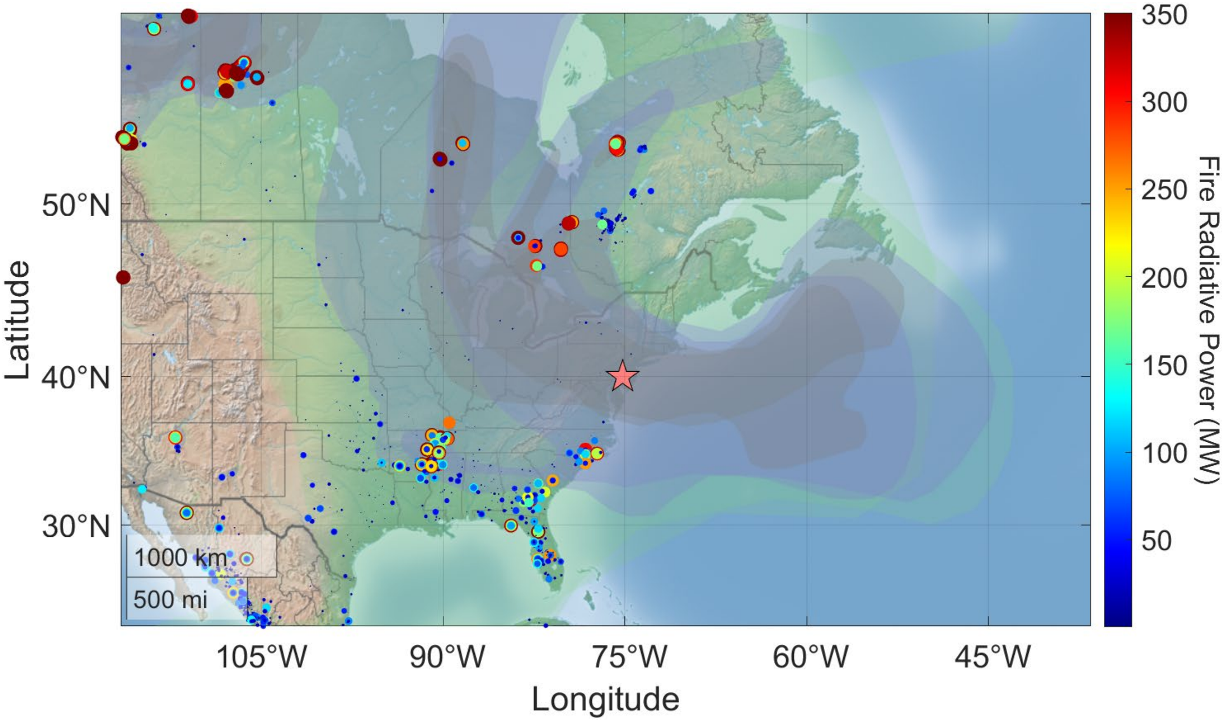

Figure S1 Surface level biomass burning smoke (colored polygons) and active fire detections (markers) on 6 June 2023 during the historic Canadian wildfire season that impacted much of the contiguous United States. This satellite-based data was acquired using MODIS Hazard mapping system[1] and was representative of the Summer 2023 biomass burning impacted periods. The translucent smoke polygons are colored green, blue, and grey for their designation of "light", "medium", and "heavy" smoke, respectively, within Hazard Mapping System. The ArcGIS (ESRI) 'color terrain' stock was used as the basemap. The Drexel University sampling site is represented by the star.

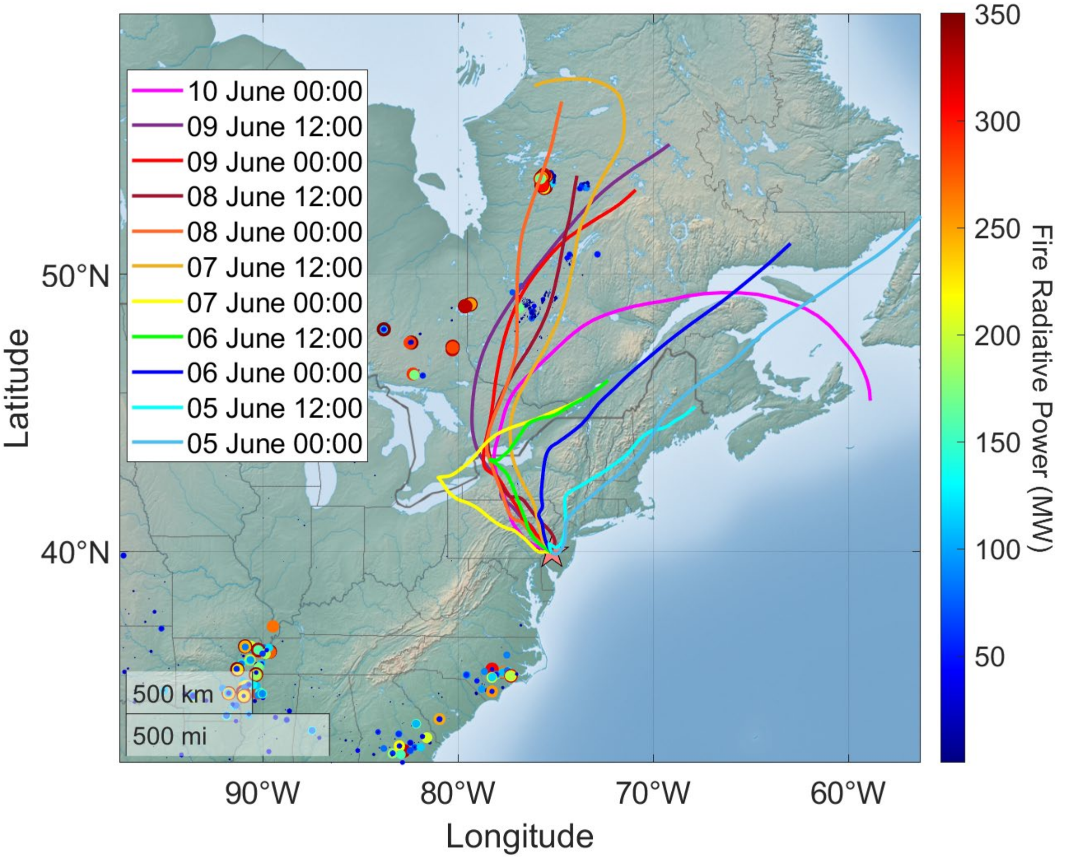

Figure S2 Fire detections (markers) on 6 June 2023 during the historic Canadian wildfire coupled with 72 hour HYSPLIT back trajectories. The legend indicates dates and times (in UTC) for each trajectory that moves backward in time 72 hours. Observations indicate the site was continually impacted by smoke between the night of 6 June and much of 9 June, consistent with the north and northwest bound trajectories here. The 'color terrain' basemap stock image (same as Fig. S1) was acquired from ArcGIS (ESRI). The Drexel University sampling site is represented by the star.

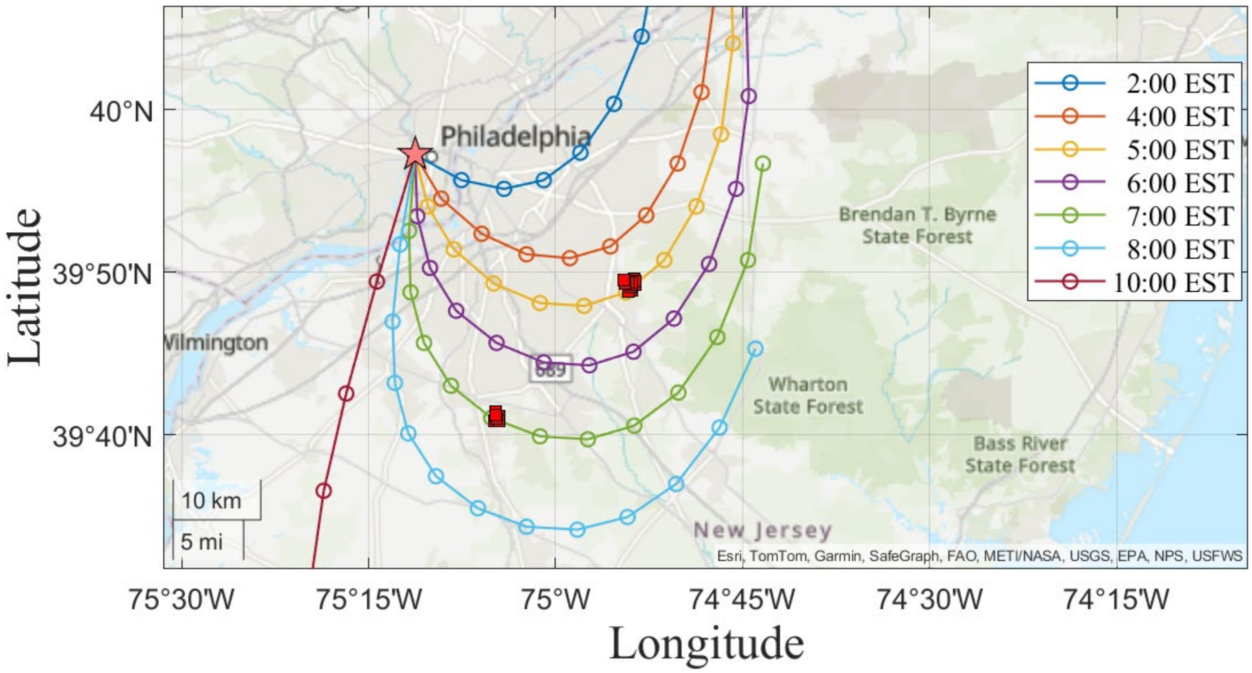

Figure S3 Local New Jersey wildfires (red markers) active during 10 November 2024 event and coinciding HYSPLIT back trajectories (NOAA, ARL). Trajectories were initialized at the Philadelphia sampling location (indicated by the star symbol) and protrude outward 12 hours. Markers along trajectories each represent 1 hour in time.

S2.0 Chemical Ionization Mass Spectrometry

S2.1 Peak fitting

Peaks are fit and integrated using TofWare (ToFWerk AG). HMHP is notably separated from the overlapping HNO_3 iodide adduct as mentioned in the main text. Figure S4 shows the peaks fit between 190.75 and 191.05 mass to charge ratio during a representative biomass burning period during Fall 2024 ambient sampling in Philadelphia. The sulfur dioxide-iodide adduct was also detected at this nominal mass during somesmoke episodes. This peak is of lesser concern due to its larger mass difference with HMHP (our main analyte of interest) and its general absence during other ambient and chamber-based observations.

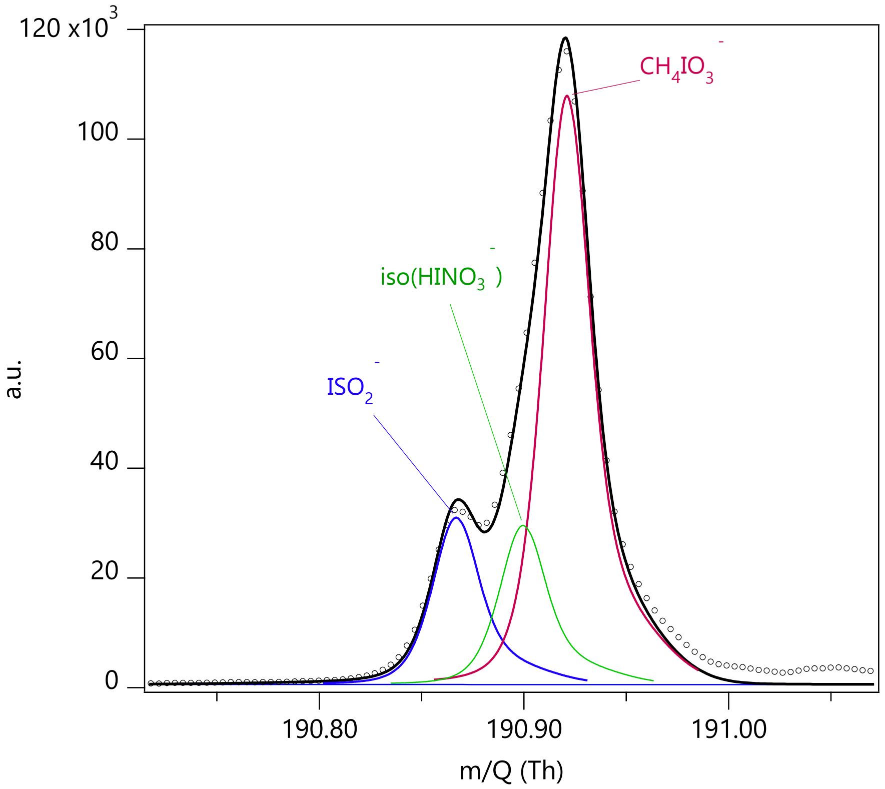

Figure S4 Representative high-resolution mass spectrum at nominal mass-to-charge ratio 191 during an ambient sampling smoke period in November 2024. Spectra were acquired at 1 Hz and are shown here averaged to approximately 15 min. The HMHP iodide adduct ($CH_4IO_3^-$) is computationally separated from the other designated ions ($IH^{15}NO_3^-$ and ISO_2^-) using tracked mass calibration data and characterized peak width and shape.

S2.2 General Calibration Information

Iodide CIMS calibration factors vary for each compound. The sensitivity is also impacted by and dependent on water vapor concentration within the CIMS IMR. The presence of water vapor produces its iodide adduct $I(H_2O)^-$, which neutral H_2O and $I(H_2O)^-$ complicate ionization chemistry through ligand-switching interactions with analyte X and its iodide adduct $I(X)^-$. Many compounds show an exponential decline in sensitivity S with increased water vapor $[H_2O]_{IMR}$ and are hence fit using the following equation:

$$S = A \cdot \exp(B \cdot [H_2O]_{IMR}) + C \quad \text{Equation S1}$$

Exponential equations have regularly been used to characterize H_2O impact on I^- CIMS sensitivity. Compounds that exhibit a lesser-dependence on water vapor or show increase in sensitivity with IMR $[H_2O]$ are often fit using polynomial equations. For reference, the primary CIMS $[H_2O]_{IMR}$ was monitored by a Vaisala HMP60 probe that sampled IMR exhaust, while the secondary EUPHORE CIMS $[H_2O]_{IMR}$ values were estimated using normalized $I(H_2O)^-$ signal.

Characterized calibration fit parameters for compounds of interest in this manuscript are included in Table S1. Sensitivity to formic acid is unique to the other compounds in that its dependence on water vapor does not exhibit the exponential decay of Equation S1. Rather a quadratic expression is used (Equation S2), and it exhibits maximum sensitivity under moderate absolute humidity values within the IMRconditions.

$$S = A \cdot [H_2O]^2 + B \cdot [H_2O] + C \qquad \text{Equation S2}$$

Table S1 Calibration fit parameters for equations. For reference, the equation parameters are fit using mole fraction of H_2O within the IMR (relevant to variable B).

Compound	A	B	C	comment
HCN	0.226	-389	0.02	
HMHP	24.2	-139	-3.35	
Formic Acid	-36534	263.9	2.94	Quadratic Formula
Acetic Acid	1.16	-487	0.096	

Temperature also influences sensitivity due to effects on ion-molecule reaction rates (e.g., $I(H_2O)^- + HMHP \rightarrow I(HMHP)^- + H_2O$).[5-7] Temperature correction was required only for the Meridian, Idaho, dataset, where the CIMS instrument and IMR experienced unstable temperatures with an overall 10 ˚C range due to the lack of IMR temperature control in combination with high variability in laboratory temperature. Temperature influence on sensitivity was previously

characterized for HCN and HONO via laboratory calibration experiments and for several organic acids using their signal responses for automated standard additions at the various CIMS temperatures of the Meridian experiment.[6, 8] Sensitivities for most analytes decrease by 3-8% ˚C^{-1}, depending on the compound, consistent with the literature.[7] A temperature dependence of 5% ˚C^{-1} was assumed for HMHP.

S2.3 HMHP Calibration and Uncertainty

The generated HMHP concentration is determined using the knowledge of the C_2H_4 + O_3 mechanism considering the rate coefficients and yields in combination with the reaction chamber residence time. Relevant reactions and their rate coefficients are included in Table S2. Here, we show the amount of HMHP generated with time using an explicit mechanism and a simplified mechanism for the experimental calibration conditions described in Sect. S2.3.1 where [C_2H_4], [O_3], and [H_2O] were in concentrations of 23.1 ppmv, 192 ppbv, and 5.0 ppthv, respectively. The explicit mechanism includes the concentration of the water dimer [$(H_2O)_2$] as 1.25 ppmv (3.06 x 10^{13} molecules cm^{-3}) that was assumed using a dimer-monomer equilibrium constant of 0.05.[9, 10]

The explicit mechanism involves the kinetics of the initial C_2H_4 + O_3 reaction, the stabilization of nascent Criegee radical CH_2OO^*, a quick conversion of CH_2OO^* to stabilized CH_2OO, and reactions of stable CH_2OO with $(H_2O)_n$. The simplified mechanism employs the kinetics of the rate determining step only (i.e., the initial reaction O_3 + C_2H_4) in combination with the HMHP yield. The overall HMHP yield in this case requires the effective HMHP yield of CH_2OO + $(H_2O)_n$ which varies with considerably with humidity. The reaction with the H_2O and $(H_2O)_2$ have unique HMHP yields being 0.73 and 0.40, respectively.[11] The effective first order rate constants for CH_2OO reacting with H_2O and $(H_2O)_2$ are 35 and 211 s^{-1}, respectively. indicating the

water dimer reaction to have more general importance in this oxidation scheme. The effective yield of HMHP + $(H_2O)_n$ in this case is 0.45 as determined by weighting yields of the two pathways by their relative importance.

We deem the use of this simplified mechanism appropriate through comparative results (Fig. S5). This simplified method may be advantageous as computing time is reduced. The knowledge of reaction rate coefficients other than the rate determining step is also not required. The effective HMHP yield must carefully be considered especially in regards to the CH_2OO + $(H_2O)_n$ reactions.

Concentrations within the IMR are known using the total reaction time. In this representative case, the reaction time is 16.4 seconds and indicated on Fig. S5 equating to a concentration of 560 pptv. The contents of the reaction chamber after 16.4 s is diluted to 247 pptv and rapidly transported to the CIMS IMR.

We ascribe an accuracy of 64% to the delivered HMHP concentration. As mentioned in the main text, the accuracy is generously estimated and accounts error in the rate coefficient of C_2H_4 + O_3 (10 %), initial C_2H_4 and O_3 concentrations (5 % each), total reaction time (2% equivalent to 0.3 s inaccuracy), and the HMHP mechanism yield (62 %). The final mentioned 62% uncertainty of the mechanism yield dominates the total uncertainty. Its value arises from the uncertainty in the 0.42 SCI yield of CH_2OO (assigned 50%) combined with the uncertainty in the weighted HMHP yield for the CH_2OO + $(H_2O)n$ reactions (assigned 37%). The SCI yield uncertainty stems from the considerable range in reported CH_2OO SCI yields for terminal alkene ozonolysis, which ranges up to ~0.6,[12] corresponding to the 50 % assigned value for the recommended 0.42 value. The 37% uncertainty mentioned assigned to the CH_2OO + $(H_2O)n$ reactions account for the errors in each individual reaction including the measured $[H_2O]$ and quantified $[(H_2O)_2]$ values, their respective

rate coefficients, and their respective HMHP yields of 0.73 and 0.40 using assigned of errors 32% and 40%, respectively). For reference, these HMHP branching yield uncertainties were assigned to generously cover literature ranges and to overlap with higher end HMHP branching yield values (e.g., Sheps et al., 2017 reported a 0.55 HMHP yield for the $(H2O)_2 + CH_2OO$ reaction).[13] We also account for error in the monomer-dimer branching ratio for $CH_2OO + (H_2O)n$, which under the representative conditions is 0.14:0.86, assuming a possible absolute error of 0.10 in each pathway (e.g., shifted branching ratio to 0.24:0.76) corresponding to relative errors of 71% and 12%, respectively. The absolute error in each $CH_2OO + (H_2O)_n$ pathway is combined in quadrature, totaling to the stated 37% relative error.

Table S2 Reaction kinetic information for relevant HMHP reactions from the ethylene-ozonolysis scheme.

Reaction	Rate Constant<br>k (298 K)	Units / Comments
$C_2H_4 + O_3 \rightarrow CH_2OO^* + HCHO$	1.6×10^{-18}	molecules^{-1} cm^3 s^{-1} (JPL)
$CH_2OO\cdot^* + M \rightarrow CH_2OO\cdot + M$	1×10^5	Units: s^{-1}<br>Rate constant was assumed<br>CH_2OO Yield: 0.42
$CH_2OO + H_2O \rightarrow CH_2(OH)(OOH)$<br>$CH_2OO + (H_2O)_2 \rightarrow CH_2(OH)(OOH) + H_2O$	2.8×10^{-16}<br>6.8×10^{-12}	Units: molecules^{-1} cm^3 s^{-1}<br>HMHP Yield: 0.73<br>HMHP Yield: 0.40

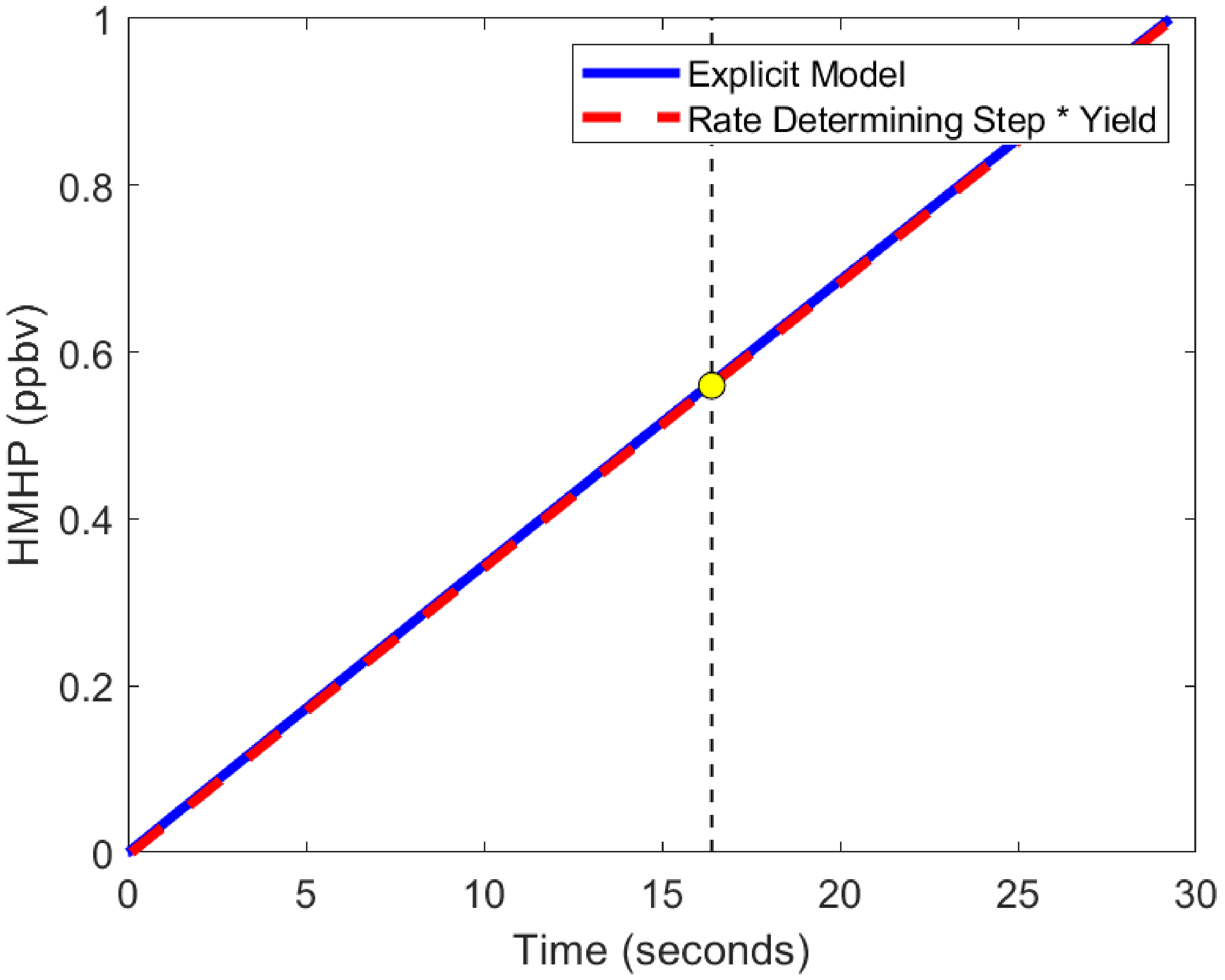

Figure S5 Comparison between the utilized rate determining step and full explicit chemical kinetic model for predicting HMHP concentration. This example matches the initial [O_3] and [C_2H_4] conditions used for obtaining calibration results in the previous section. The dashed vertical represents the residence time within the reaction chamber. A marker is included at the residence time-HMHP concentration.

S2.3.2 Relationship between $I(H_2O)^-$ and $[H_2O]_{IMR}$

The absolute humidity was not monitored for the secondary CIMS instrument's IMR used during EUPHORE chamber experiments. We instead rely on the normalized $I(H_2O)^-$ signal for sensitivity. The ratio (i.e., $I(H_2O)^-/[I^- + I(H_2O)^-]$) during gas-phase sampling was 0.291. The sensitivity-absolute humidity relationship plot (main text Fig. 4) is updated to include this $I(H_2O)^-$ metric in Fig. S6. The humidity-relationship suggests a 5.5 ppthv value for $[H_2O]_{IMR}$ along with

an effective CIMS sensitivity to HMHP of 8.6 ncps ppthv^{-1}. Results for HCN are also presented. The effective sensitivity to HCN at 5.5 ppthv is 0.047 ncps pptv^{-1}.

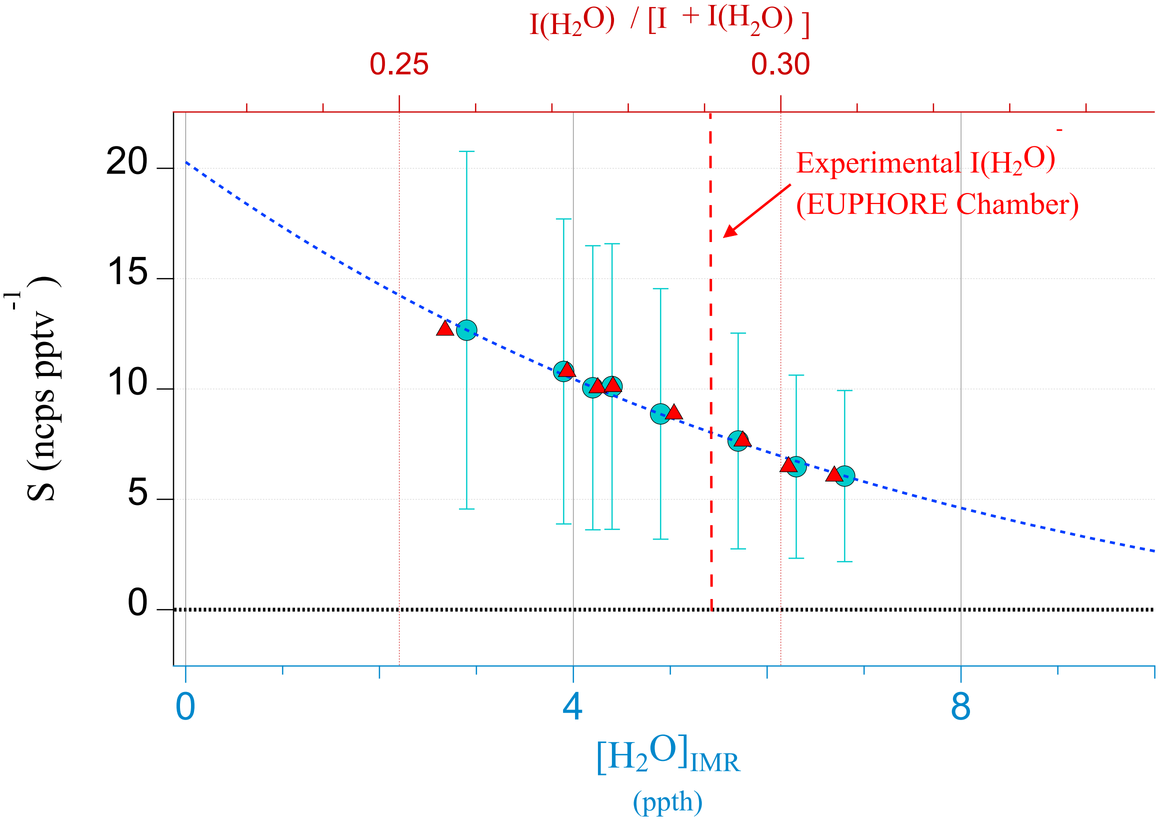

Figure S6 HMHP sensitivity data plotted against normalized I(H$_2$O)$^-$ (red triangles; top axis) overlaid onto the Fig. 3 main text sensitivity results (plotted against [H$_2$O]$_{IMR}$; blue markers)

References

(1) NOAA. *Hazard Mapping System Fire and Smoke Product*. https://www.ospo.noaa.gov/products/land/hms.html#data (accessed 2025 08/01).

(2) Stein, A. F.; Draxler, R. R.; Rolph, G. D.; Stunder, B. J.; Cohen, M. D.; Ngan, F. NOAA's HYSPLIT atmospheric transport and dispersion modeling system. *Bulletin of the American Meteorological Society* **2015**, *96* (12), 2059–2077.

(3) Mitman, H. *Smoky conditions set off CO alarms, wake residents near Bethany Run Wildfire*. 2024. (accessed 2025 6/13).

(4) NASA. *Fire Information for Resource Management System*. https://www.earthdata.nasa.gov/data/tools/firms (accessed 2026 01/24).

(5) Lindsay, A. J. Non-combustion Emissions of Organic Acids at a site near Boise, Idaho. *EST Air* **2024**, *under review*.

(6) Lindsay, A. J. W., Ezra C; Banecker, Kyle; Weesner, Brigitte M. Vehicle Emission Ratios of HCN and HONO measured near a highway in Meridian, Idaho. *under review* **2025**.

(7) Robinson, M. A.; Neuman, J. A.; Huey, L. G.; Roberts, J. M.; Brown, S. S.; Veres, P. R. Temperature-dependent sensitivity of iodide chemical ionization mass spectrometers. *Atmospheric Measurement Techniques* **2022**, *15* (14), 4295–4305.

(8) Lindsay, A. J.; Weesner, B. M.; Banecker, K.; Feinman, L. V.; Long, R. W.; Landis, M. S.; Wood, E. C. Noncombustion Emissions of Organic Acids at a Site near Boise, Idaho. *ACS Es&t Air* **2024**, *1* (12), 1568–1578.

(9) Ruscic, B. Active thermochemical tables: Water and water dimer. *The Journal of Physical Chemistry A* **2013**, *117* (46), 11940–11953.

(10) Chao, W.; Hsieh, J.-T.; Chang, C.-H.; Lin, J. J.-M. Direct kinetic measurement of the reaction of the simplest Criegee intermediate with water vapor. *Science* **2015**, *347* (6223), 751–754.

(11) Nguyen, T. B.; Tyndall, G. S.; Crounse, J. D.; Teng, A. P.; Bates, K. H.; Schwantes, R. H.; Coggon, M. M.; Zhang, L.; Feiner, P.; Milller, D. O.; et al. Atmospheric fates of Criegee intermediates in the ozonolysis of isoprene. *Physical Chemistry Chemical Physics* **2016**, *18* (15), 10241–10254.

(12) Cox, R. A.; Ammann, M.; Crowley, J. N.; Herrmann, H.; Jenkin, M. E.; McNeill, V. F.; Mellouki, A.; Troe, J.; Wallington, T. J. Evaluated kinetic and photochemical data for atmospheric chemistry: Volume VII–Criegee intermediates. *Atmospheric Chemistry and Physics Discussions* **2020**, *2020*, 1–41.

(13) Sheps, L.; Rotavera, B.; Eskola, A. J.; Osborn, D. L.; Taatjes, C. A.; Au, K.; Shallcross, D. E.; Khan, M. A. H.; Percival, C. J. The reaction of Criegee intermediate CH 2 OO with water dimer: primary products and atmospheric impact. *Physical Chemistry Chemical Physics* **2017**, *19* (33), 21970–21979.
